# Mini-midvastus total knee arthroplasty does not result in superior gait pattern

**DOI:** 10.1007/s00167-014-3154-7

**Published:** 2014-07-04

**Authors:** M. C. Liebensteiner, M. Thaler, J. M. Giesinger, S. Fischler, D. C. Coraça-Huber, M. Krismer, E. Mayr

**Affiliations:** 1 Department of Orthopaedic Surgery, Innsbruck Medical University, Anichstrasse 35, 6020 Innsbruck, Austria; 2 Department of Psychosocial Research and Epidemiology, The Netherlands Cancer Institute, Amsterdam, The Netherlands; 3 Department for Experimental Orthopaedics, Innsbruck Medical University, Innsbruck, Austria; 4 Department of Orthopaedic Surgery, Celle General Hospital, Celle, Germany

**Keywords:** Total knee replacement, Ambulation, Walking patterns, Mini-midvastus approach

## Abstract

**Purpose:**

Previous studies dealing with gait after minimally invasive surgery (MIS) total knee arthroplasty (TKA) are rare and insufficient. It was the purpose of the study to determine in a prospective, comparative setting whether MIS influences the outcome of TKA in terms of typical 3D gait parameters.

**Methods:**

Patients scheduled for TKA or MIS TKA were invited to participate. MIS TKA was defined as TKA with shorter skin incision, mini-midvastus arthrotomy, special instruments, and avoidance of tibiofemoral dislocation and patella eversion. All other intra- and perioperative aspects were identical for both groups. A 3D gait analysis was performed with a VICON system 1 month preoperative and 8 weeks post-operative. A multivariate analysis of variance was conducted including the main effects time (pre- and post-surgery) and surgical group and the group-by-time interaction effect.

**Results:**

Seventeen MIS TKA patients and 20 TKA patients were eligible for the final analysis. We determined neither inter-group differences nor time × group interactions for any gait variables (temporospatial, ground reaction forces, joint angles and joint moments)—except for the varus–valgus knee kinematics. In pre- to post-operative comparison, the maximum valgus sway increased in the MIS group, whereas it decreased in the conventional group (*p* = 0.001).

**Conclusion:**

From our findings, it was concluded that MIS TKA does not result in a superior walking pattern 8 weeks post-operative. Because we previously also observed mini-midvastus MIS TKA to have equal or slightly inferior results with regard to knee scores, knee torque, radiographic outcome and tourniquet/operating time, we discontinued the procedure.

**Level of evidence:**

Prospective comparative study, Therapy, Level II.

## Introduction

Minimally invasive surgical (MIS) total knee arthroplasty (TKA) was developed to facilitate early and intermediate-term rehabilitation. MIS generally includes reduced length of skin incision, less invasive arthrotomy, avoidance of tibiofemoral dislocation or hyperflexion, and use of special cutting blocks and retractors [2, 5].

The literature contains inconsistent findings as to whether MIS achieves its stated goals. For example, in terms of typical knee scores, some authors reported superior results for MIS TKA [2, 4, 7], whereas others found outcomes to be similar for MIS TKA and conventional TKA [12, 13, 15].

Given the less invasive dissection of the extensor apparatus, MIS TKA might also be associated with better post-operative gait characteristics. Only few authors have dealt with this issue so far. One research group found superior gait pattern among MIS patients [1, 9], but their studies dealt only with *navigated* MIS TKA and failed to analyse kinetic gait data. Conflicting findings were reported by Satterly et al. [16] in a recent publication. Comparing the TKA approaches, midvastus, subvastus and mini-parapatellar with the standard parapatellar approach, the authors stated that no approach was superior with regard to gait characteristics. Similarly, Wegrzyn et al. [18] reported no advantages in gait characteristics for mini-subvastus MIS TKA as compared to conventional TKA. Also, Nestor et al. [15] compared MIS and standard TKA with regard to gait characteristics and reported no effect of the surgical approach. However, that study failed to analyse kinetic and kinematic gait data.

To the best of our knowledge, only few studies to date have investigated potential effects of MIS TKA on gait characteristics. While some researches investigated only *navigated* MIS TKA [1, 9, 16], others failed to determine a full set of gait parameters (kinetics, kinematics) [15]. Only Wegrezyn et al. investigated gait after standard TKA versus MIS TKA (mini-subvastus) and reported no advantage for the MIS procedure.

Given conflicting reports in the literature, it was the aim of our study to determine in a prospective, comparative setting whether MIS influences the outcome of TKA in terms of typical 3D gait parameters. As MIS TKA claims to apply a less invasive dissection of the extensor apparatus, we expected that this would result in, e.g. faster walking speed, less double support time, higher vertical ground reaction force, improved sagittal knee ROM. To know whether these ideas are true might influence orthopaedic surgeons’ decisions whether to do or not to do MIS in TKA (together with other outcome parameters such as revision rate, score outcome and quality of life).

It was hypothesized that MIS would affect temporospatial parameters (H1), ground reaction forces (GRF) (H2), knee kinematics (H3) and knee kinetics (H4). It was also planned to investigate kinematic and kinetic variables of joints other than the knee, but these were defined as exploratory and therefore not linked to a hypothesis.

## Materials and methods

**Fig. 1:**
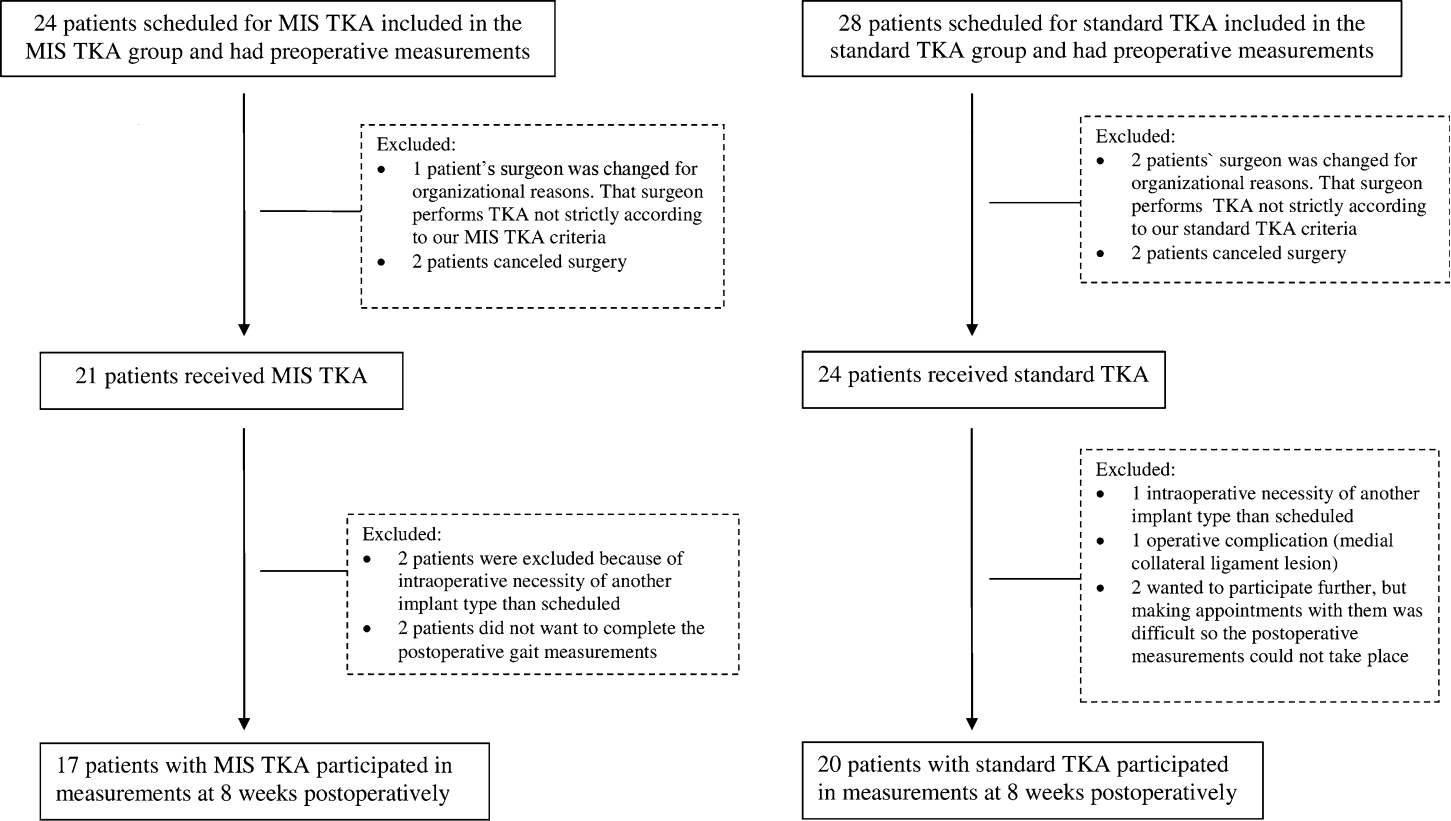
Patient flow

Applying a prospective, comparative study design, consecutive patients with osteoarthritis on the waiting list for TKA were included. Exclusion criteria were (1) age younger than 55 years or older than 80 years, (2) neuromuscular or neurodegenerative disease, (3) prior arthrodesis in any joint of the lower limbs (except for toes II–V), (4) prior TKA on the contralateral side, (5) prior arthroplasty of the ipsilateral hip or ankle and (6) constant need for walking aids. Patient flow is detailed in Fig. 1.

Patient positioning, antibiotic and deep vein thrombosis prophylaxis, draping and tourniquet control were standardized, and identical cruciate-retaining TKAs were performed in both groups (Scorpio™; Stryker Corp, Kalamazoo, MI, USA) using intramedullary referencing in the femur and extramedullary referencing in the tibia. In accordance with the clinical routine at our institution, the patella was left unresurfaced.

In the standard TKA group, a midline skin incision and a standard medial parapatellar arthrotomy were performed and the patella was everted. The prosthesis was implanted according to the manufacturer’s instructions using a measured resection technique and standard cutting blocks and instruments.

In the MIS TKA group, a midline skin incision was followed by a medial mini-midvastus arthrotomy (1–3 cm) [5]. The patella was subluxated instead of being everted. Special downsized retractors and cutting jigs [2] were used in accordance with the operation manual for the Scorpio™ MIS procedure, as provided by the manufacturer. The major differences as compared to standard TKA were less invasive arthrotomy, absence of patella eversion, use of special instruments and the fact that the tibiofemoral articulation was dislocated or hyperflexed only during cementing of the tibia [2, 5].

All patients underwent the same standardized rehabilitation programme after surgery. Patients were mobilized from the second post-operative day under supervision of our physiotherapists. Exercises included continuous passive motion, assisted and unassisted knee extension, walking and stair-climbing with two crutches, and progression as tolerated.

Preoperative data were collected 1 month before surgery, and post-operative data were collected 8 weeks post-operatively. 3D gait analysis was performed preoperatively and 8 weeks post-operative with a 3D motion analysis system (VICON, Oxford, UK and AMTI, Watertown, MA, USA) applying a 4-segment lower-body marker model. During level walking at self-selected speed temporospatial parameters, joint angles (kinematics), external joint moments (kinetics) and GRFs were determined with the software packages of the manufacturer of the motion analysis system (Workstation V4.6 and Polygon Authoring Tool V3.1; VICON, Oxford, UK). The accuracy of our measuring system was previously tested [19]. This study shows that with dynamic calibration, overall accuracy was 63 ± 5 µm.

The study protocol was approved by the ethics committee of our medical university, and written informed consent was obtained from all patients before inclusion in the study. The rights of the subjects were protected.

### Statistical analysis

Sample characteristics are given as means, standard deviations, range and frequencies. To analyse the impact of MIS on 3D gait parameters, we used a multivariate analysis of variance (MANOVA) including the main effects time (pre- and post-surgery) and surgical group and the group-by-time interaction effect. The gait parameters (i.e. the dependent variables) were grouped according to the hypotheses H1 to H4 and analysed separately. To determine the significance of the multivariate tests, we used the Hotelling-Spur statistics. Greenhouse–Geisser correction was applied if the sphericity assumption was not met in univariate testing. All analyses were performed with SPSS 20.0.

Power analysis was done for a group-by-time interaction in a repeated measure analysis of variance including two groups and two time points (alpha = 0.05, beta = 0.20). The interaction term reflects differences with regard to the change in gait pattern between the two groups. A sample size of 20 patients per group (40 in total) is sufficient to detect an interaction effect of Cohen’s *f* = 0.45 [3].

## Results

Pre- and post-operative participant characteristics are detailed in Table 1.

**Table 1:** Participant characteristics presented as means and standard deviations

	MIS TKA (*n* = 17)	Standard TKA (*n* = 20)	*p* value
Age (year)	66.4 ± 5.0	68.2 ± 7.2	n.s.
Height post-operative (month)	1.66 ± 0.08	1.65 ± 0.08	n.s.
Weight post-operative (kg)	81.3 ± 13.5	83.4 ± 11.5	n.s.
BMI post-operative (kg/m^2^)	29.5 ± 3.8	30.7 ± 3.5	n.s.
Gender			
Female	11	11	n.s.
Male	6	9	
Side			
Left	9	7	n.s.
Right	8	13	

*BMI* body mass index, *TKA* total knee athroplasty, *MIS* minimally-invasive surgery

For the temporospatial parameters, we found the factor ‘surgical group’ to have no influence. However, we observed a significant pre-to-post-operative increase in stride length for both groups (*p* = 0.031). There were no time × group interactions (H1).

Analysis of the vertical component of the GRF did not reveal any influence of the factors ‘surgical group’ or ‘time’, nor were there time × group interactions for any of the three components of GRF (H2).

For sagittal knee kinematics, neither the surgical group nor the factor time was seen to have an influence, nor were there time × group interactions. Frontal knee kinematics showed significant time × group interactions for the maximum valgus during gait (*p* = 0.001): In pre-to-post-operative comparison, the maximum valgus *in*creased in the MIS group, whereas it *de*creased in the conventional group (H3).

Sagittal knee moments (extensor and flexor moment) were affected neither by the surgical group nor by the time. No time × group interactions were observed. Similarly, there were no group differences or time × group interactions in knee moments in the frontal plane (H4).

(For detailed results, see Tables 2 and 3).

**Table 2:** Descriptive statistics of temporospatial and kinematic gait parameters

	Unit	MIS TKA				TKA			
		Pre		Post		Pre		Post	
		Mean	SD	Mean	SD	Mean	SD	Mean	SD
Temporospatial parameter									
Gait velocity	m/s	0.9	0.2	0.9	0.2	0.8	0.2	0.9	0.2
Stance	% gait cycle	60.8	2.6	60.5	2.1	62.3	4.1	61.4	2.4
Swing	% gait cycle	39.2	2.6	39.5	2.1	37.7	4.1	38.7	2.4
Double support	s	0.3	0.1	0.3	0.1	0.3	0.2	0.3	0.1
Double support	% gait cycle	24.0	6.1	22.1	4.1	25.8	8.8	23.9	4.5
Stride length	m	1.1	0.2	1.1	0.1	1.0	0.2	1.0	0.1
Cadence	steps/min	102.7	13.1	102.3	8.0	101.4	11.7	102.3	11.3
Step width	m	0.1	0.0	0.1	0.0	0.2	0.0	0.2	0.0
Gait cycle duration	s	1.2	0.2	1.2	0.1	1.2	0.2	1.2	0.1
Kinematics									
Sagittal									
Sagittal knee angle (+ values: flexion)									
Max knee flexion stance		19.2	7.8	21.5	5.5	20.0	8.0	18.3	6.9
Max knee flexion swing		56.7	11.0	58.7	6.3	52.2	9.1	52.3	7.0
Min knee flexion gait cycle		12.5	6.8	15.3	4.3	13.4	8.1	13.0	6.2
Knee flexion at toe off		33.9	7.3	3.36	5.2	34.4	7.3	34.1	5.2
Knee flexion at foot strike		14.5	6.2	15.9	4.3	14.3	7.5	12.5	5.6
Total sagittal knee ROM gait cycle		45.1	11.7	44.9	6.8	40.3	10.4	41.2	7.9
Sagittal hip angle (+ values: flexion)									
Max hip flexion gait cycle		32.4	6.5	34.8	5.9	37.1	6.7	35.6	6.7
Min hip flexion gait cycle		−5.4	6.7	−4.0	7.4	1.8	9.8	−1.6	6.7
Total sagittal hip ROM gait cycle		37.7	5.2	38.8	4.0	35.3	7.9	37.2	4.8
Sagittal ankle angle (+ values: dorsiflexion)									
First minimum gait cycle		−1.7	10.6	−3.3	3.8	−1.9	7.6	−4.6	4.1
Maximum gait cycle	deg	17.0	10.3	16.3	3.4	16.7	6.9	16.5	3.3
Second minimum gait cycle		−4.6	12.1	−6.0	6.6	−2.6	9.9	−4.3	7.3
Total sagittal ankle ROM gait cycle		23.3	5.0	23.0	3.9	21.2	5.7	22.8	4.8
Frontal									
Frontal pelvis angle (pelvic obliquity) (+ values: up)									
Maximum gait cycle		1.7	2.7	1.4	2.7	2.2	3.3	2.8	2.8
Minimum gait cycle		−2.8	2.4	−2.6	2.1	−2.3	2.9	−1.9	2.1
Total frontal pelvis ROM gait cycle		4.5	2.0	4.0	2.2	4.5	2.2	4.6	2.4
Frontal hip angle (+ values: abduction)									
Maximum gait cycle		6.5	5.7	9.7	3.8	7.4	5.9	7.4	4.0
Minimum gait cycle		−0.8	6.1	2.6	4.3	−0.1	6.6	0.1	4.3
Total frontal hip ROM gait cycle		7.3	3.5	7.1	3.4	7.5	3.6	7.3	3.6
Frontal knee angle (+ values: varus)									
Maximum stance		8.5	5.8	5.0	5.0	7.4	8.5	6.9	6.4
Minimum stance		−2.6	5.1	−6.3	4.0	−5.8	6.9	−4.4	5.0
Total frontal knee ROM stance		11.0	4.6	11.3	5.0	13.2	5.7	11.2	6.2

**Table 3:** Descriptive statistics of kinetic gait parameters and ground reaction forces

	Unit	MIS TKA				TKA			
		Pre		Post		Pre		Post	
		Mean	SD	Mean	SD	Mean	SD	Mean	SD
Ground reaction forces									
GRF vertical (Fz) (+ values: up)									
Fz1: first maximum	N/kg	9.6	0.4	9.6	0.2	9.7	0.4	9.5	0.3
Fz2: first minimum	N/kg	8.6	0.6	8.7	0.4	8.7	0.5	8.7	0.3
Fz3: second maximum	N/kg	9.9	0.6	9.9	0.4	9.7	0.5	9.7	0.4
Fz1-time: time to Fz1	% stance	31.1	7.4	30.3	5.1	31.2	7.4	31.6	6.4
Fz2-time: time to Fz2	% stance	48.7	6.6	49.4	5.9	51.5	8.8	51.3	6.9
Fz3-time: lime to Fz3	% stance	70.5	8.4	74.2	5.0	74.7	4.9	73.7	3.3
GRF ap shear (Fx) (+ values: anterior)									
Fx1: minimum	N/kg	−0.8	0.2	−1.0	0.2	−1.0	0.4	−0.9	0.2
Fx2: maximum	N/kg	1.1	0.4	1.3	0.4	1.1	0.4	1.2	0.3
Fx1-time: Time to Fx1	% stance	16.8	5.5	16.7	6.9	16.6	6.0	15.7	5.2
Fx2-time: Time to Fx2	% stance	86.1	4.5	89.1	1.9	85.6	6.2	87.8	3.1
GRF ml shear (Fy) (+ values: lateral)									
Fy1: first minimum	N/kg	−0.4	0.2	−0.4	0.1	−0.5	0.1	−0.5	0.1
Fy1-time: time to Fy1	% stance	31.2	6.6	33.0	4.9	30.4	7.8	31.6	5.7
Kinetics (internal joint moments)									
Sagittal									
Sagittal hip moment (+ values: extensor)									
Maximum gait cycle		0.7	0.2	0.8	0.2	0.8	0.3	0.8	0.2
Minimum stance		−0.3	0.2	−0.3	0.2	−0.3	0.1	−0.3	0.1
Minimum swing		−0.2	0.1	−0.3	0.1	−0.3	0.1	−0.3	0.1
Sagittal knee moment (+ values: extensor)									
Maximum gait cycle		0.2	0.1	0.3	0.1	0.2	0.1	0.2	0.1
Minimum stance		−0.3	0.1	−0.2	0.1	−0.2	0.1	−0.2	0.1
Minimum swing	Nm/kg	−0.2	0.1	−0.2	0.1	−0.2	0.0	−0.2	0.0
Sagittal ankle moment (+ values: plantarflexion)									
Maximum gait cycle		1.3	0.3	1.3	0.2	1.3	0.3	1.3	0.2
Frontal									
Frontal hip moment (+ values: abduction)									
Maximum stance		0.9	0.2	1.0	0.2	0.9	0.2	0.9	0.2
Frontal knee moment (+ values: abduction)									
Maximum stance		0.5	0.2	0.3	0.2	0.5	0.2	0.4	0.1
Minimum stance		−0.1	0.0	0.0	0.0	−0.1	0.1	0.0	0.0

Beyond the hypotheses also no significant group differences were found for joint angles or joint moments of the hip or ankle.

## Discussion

As the most important finding of our study, MIS was seen to not result in a superior walking pattern 8 weeks after TKA. Most gait patterns showed no significant differences between groups, except for inferior results in MIS patients regarding maximum valgus kinematics.

An attempt to integrate our results in the findings made in previous research revealed that the specific issue of ‘gait characteristics of MIS versus standard TKA’ was only rarely dealt with. Two months post-operative Wegrzyn et al. [18] compared gait in mini-subvastus MIS TKA and standard TKA patients. Similar to our findings, they observed no advantages for the MIS procedure. Also, Nestor et al. [15] investigated gait after mini-midvastus MIS TKA versus standard TKA and found no differences between the groups. However, no comprehensive gait analysis was performed because kinetic and kinematic gait data were not assessed. Satterly et al. [16] investigated the effect of four different surgical approaches in navigated TKA (medial parapatellar, mini-medial parapatellar, medial subvastus and mini-midvastus) with regard to gait characteristics. They reported that none of those approaches showed a superior outcome with regard to gait. However, the results of that study might be of less relevance for the specific issue at hand, because the authors (a) analysed *navigated* TKA and (b) did not report having investigated ‘strict’ MIS TKA as previously defined [2, 5]. Our findings stand in contrast to those of a research group that found superior gait pattern in MIS patients [1, 9]. However, again the authors investigated *navigated* MIS TKA, which is a slightly different issue, and failed to collect kinetic gait data. In summary, only the above-mentioned study by Wegrzyn et al. [18] investigated all aspects of 3D gait analysis (temporospatial, kinematic and kinetic parameters) after non-navigated MIS TKA versus non-navigated standard TKA. The results of our current study support that recent publication.

Recently, the results of our MIS versus standard TKA population were published in terms of other outcome parameters: WOMAC scores, knee extensor/flexor torque, radiographic outcome and tourniquet/operating time [12]. We found WOMAC score, knee extensor and flexor strength to have equal results, and leg axis, component positioning and tourniquet/operating time in MIS patients to even be slightly inferior. Thus, it would seem that the concept of MIS TKA does not work in our hands. We feel that this can not be attributed to a learning curve, because MIS TKA has been routinely performed at our institution for 5 years. For this reason, we regarded ourselves as being beyond the learning curve as published by King et al. [8]. However, and also in the light of the above-mentioned studies [1, 9, 16], it can be speculated whether computer-assisted surgery could have altered our findings.

The only significant group difference of the current study—more valgus kinematics in MIS TKA—might also be discussed in the context of the above-mentioned previous publication [12]. Whole leg axis was significantly more valgus in the MIS TKA group as determined by whole leg radiographs. Those findings of static alignment are in good agreement with the valgus kinematics during gait (dynamic alignment) observed in the current study. The slightly inferior component positioning and whole leg alignment in MIS TKA were attributed to the limited surgical access [12].

It indeed might also be speculated whether such valgus leg alignment is associated with medial condylar lift-off. As previous research indicated that condylar lift-off is related to increased polyethylene wear [6, 17], we consider the valgus kinematics of our MIS TKAs to be clinically relevant.

Regardless of the issue of ‘MIS versus conventional’, there is good consensus that in most patients, TKA is advantageous in terms of pain and function. However, some gait parameters remain different from those of healthy controls. McClelland et al. [14] reviewed studies that investigated gait in TKA patients versus controls and reported as follows: TKA patients have (1) less total sagittal knee ROM, (2) less knee flexion during the swing phase, (3) less ROM during the loading phase of stance (4) abnormal (non-biphasic) knee moment pattern in the sagittal plane. Others even reported that TKA did not result in improvement of any of the kinetic or kinematic gait pattern although the patients had benefitted in terms of pain and function [10].

Therefore, it could be argued that gait analysis is not a useful tool for evaluation of TKA. In this connection, we agree with Wright who recommended a combination of a knee score, a health-related quality-of-life questionnaire and an activity score [20]. However, for some research purposes, a gait analysis might still be useful.

The following limitations of the study are acknowledged. Firstly, we did not randomize the patients because of the impracticability of persuading surgeons to modify routines with which they were comfortable. A randomized trial would have reduced the risk of bias between the groups. Secondly, we performed the gait analyses on only two occasions. More post-operative measurement would have provided additional information on the course of gait recovery. Thirdly, MIS TKAs were always performed by one of two experienced knee surgeons, whereas standard TKAs were performed by a larger pool of surgeons with varying degrees of experience. That could have exerted a favourable impact on the gait parameters in the MIS group, while in actual fact, we found no differences. In addition, it would have been of interest to test also at different walking speeds and inclinations (e.g. treadmill) and to perform further tests in the early post-operative period (e.g. after 4 weeks). As we investigated only the mini-midvastus type of MIS surgery, we cannot expand our findings to other types of MIS TKA surgery (e.g. subvastus or quadsparing).

However, the study at hand is the second publication (after Wegrzyn et al. [18]) to report on an investigation of all aspects of 3D gait analysis (temporospatial, kinematic and kinetic) after non-navigated MIS TKA versus non-navigated standard TKA. Therefore, we believe it substantially contributes to the current scientific knowledge. The strengths of the study also lie in its prospective, comparative design (Level of Evidence: 2).

The study at hand also provides clinically relevant findings. In addition to other tools (knee scores, quality-of-life scores, revision rates, etc.), gait analysis delivers important information on the outcome of TKA, especially regarding functional outcome [11].

## Conclusions

In conclusion, we did not identify superior gait characteristics in mini-midvastus MIS TKA patients 2 months post-operative. Because we previously also determined equal or slightly inferior results of mini-midvastus MIS TKA with regard to knee scores, knee torque, radiographic outcome and tourniquet/operating time, we discontinued the procedure.
